# I’m Doing Better on My Own: Social Inhibition in Vocabulary Learning in Adults

**DOI:** 10.3389/fpsyg.2019.01350

**Published:** 2019-06-05

**Authors:** Clara D. Martin, Amy Underwood, Nicola Molinaro

**Affiliations:** ^1^BCBL – Basque Center on Cognition, Brain and Language, San Sebastian, Spain; ^2^IKERBASQUE, Basque Foundation for Science, Bilbao, Spain

**Keywords:** vocabulary learning, second language acquisition, video deficit effect, social inhibition, education

## Abstract

Vocabulary learning is better achieved by children facing a teacher than when presented to the same teacher through video (so-called “video deficit” effect), which has significant implications for toddlers’ education. Since millions of adults also learn new vocabulary when acquiring a second language (L2), it is important to explore whether adults suffer from “video deficit” effects, as children do. In the present study, we report two experiments in which Spanish native late learners of English were involved in a vocabulary learning task. In Experiment 1, participants had to learn English (L2) labels associated to real objects. In Experiment 2, participants had to learn English (L2) and Spanish (L1) labels associated to novel objects. In both experiments, vocabulary learning was divided into three conditions: In the *NoFace* condition, participants were presented with the objects and their auditory labels, through video. In the *Video* condition, a teacher was showing the objects and uttering their names, through video. The *Live* condition was equivalent, except that the teacher was facing the participants in the room. Each condition was followed by a recall test. Better learning in *Video* compared to *NoFace* condition revealed that adults benefit from the teacher’s display with direct gaze, confirming the fundamental role of face display with direct gaze in social communication in adults. Interestingly, adults learned better through *Video* than in the *Live* condition. Those results were obtained in L2 vocabulary learning in both Experiments 1 and 2, and also generalized to native language in Experiment 2. We argue that adults suffer from social inhibition, meaning that they perform worse when in the presence of another person during task performance. In sum, we show that video-mediated teaching might not be detrimental for adults learning new vocabulary lists, as it is the case for young children. These results might have important implications for pedagogical programs targeting adults’ second language vocabulary learning, since proper acquisition of vocabulary list can be achieved through video including a teacher’s display.

## Introduction

### L2 Vocabulary Learning in Adults

Millions of adults acquire a foreign language during adulthood, and they usually struggle with phonology, grammar, and vocabulary learning (Ellis, 1985; Cook, 2008). There is a large literature on the factors modulating L2 acquisition, and on the way it can be improved. In the present study, we focused on vocabulary learning. Even when focusing on this restricted aspect of L2 acquisition, learning situations are multiple and difficult to control. In fact, adult learners of an L2 encounter a new L2 word several times, in several situations (e.g., in classroom, in social interactions, and in medias) and modalities (novel word can be heard, read, and produced). Furthermore, learning a new word does not imply only learning a label for an object (or translation equivalent of an L1 word) but also its meaning, proper usage in sentences, etc. In the present study, we focused on the first and basic stage of vocabulary acquisition (i.e., associating a label to an object) in a simple learning situation (i.e., one presentation of the object and associated label). There is a large literature already on acquisition of L2 vocabulary [see Schmitt (2008); Pinar (2016) and Rasouli and Jafari (2016) for reviews]. First, vocabulary learning can be intentional (explicit) or incidental (new words learned without direct attention; see for instance Nation, 2001; Schmitt, 2008). Here, we focused on intentional learning. Several studies have explored how intentional vocabulary learning can be improved, and it has been shown, for instance, that word learning success is enhanced by repetition (Webb, 2007), engagement (e.g., new words used in a writing *versus* reading task; Stirling, 2003), word usage outside of the classroom (Dewey, 2008), and immersion (Dewey, 2008; Llanes and Muñoz, 2012). We also know that several individual factors such as gender (Gu, 2005), age (Llanes and Muñoz, 2012), proficiency, and motivation (Kojic-Sabo and Lightbown, 1999) affect vocabulary learning. Here, we propose a novel approach in which we do not manipulate or explore social factors such as motivation or immersion, but social context during learning, such as interaction with the teacher. As a very first step in exploring the complex factor that is social context, we focused on whether direct interaction modulates L2 vocabulary learning in adults. The rational for exploring direct interaction in L2 vocabulary learning comes from research on language development that is described below.

### Video-Deficit Effect in Toddlers

Direct interaction, an important aspect of social situations, seems to be mandatory for efficient vocabulary learning in toddlers (Krcmar et al., 2007; DeLoache et al., 2010; O’Doherty et al., 2011). Direct interaction implies that toddlers actively participate in the learning task instead of passively observing the learning situation. Some of the most striking studies revealing the mandatory role of social direct interactions are the ones showing that children do not learn efficiently from exposure through video, the so-called “video deficit” effect (Anderson and Pempek, 2005). The “video deficit” effect is the recurrent observation that learning, behavior imitation, and instruction following is better achieved by children facing the “teacher” than when presented to the same teacher through video (see for instance Barr and Hayne, 1999; Troseth et al., 2006). Regarding vocabulary learning, which is the scope of the present study, the “video deficit” effect has been repeatedly reported. It is known that novel word learning can be achieved by 15-, 24-, and 30-month olds from an adult who is present in the room. However, the words are not learned as well from a person on video offering the same cues to the toddlers [Krcmar et al., 2007; DeLoache et al., 2010; O’Doherty et al., 2011; Roseberry et al., 2014; see also Kuhl et al. (2003) for similar “video deficit” effect in phonetic learning]. This is not to say that toddlers do not learn through video, but vocabulary learning is less efficient from television compared to direct interaction in children younger than 2 years of age. In sum, direct interaction with a teacher seems to be the ideal social situation for proper learning in young children. Importantly, it could be that the advantage in learning through direct interaction disappears or is even reversed during development. In fact, shifting behavior is observed during development when participants are facing social interactions, as revealed by the so-called “audience effect” described below.

### Audience Effects and Social Inhibition in Adults

Audience effects refer to the influence of the presence of another person (or more) on someone’s performance in a given task (Zajonc, 1965; Tennie et al., 2010). Those effects vary considerably with task difficulty, audience identity, age, etc. When considering performance in difficult tasks, in the presence of an adult observer (uttering no positive or negative judgment), different aged-related patterns tend to emerge: Performance tends to increase (or remain stable) under adult presence versus “alone” condition in 4- to 10-year old children (Meddock et al., 1971; Newman et al., 1978; Chevallier et al., 2014). A shift in behavior seems to happen during development, with 10- to 17-year old adolescents showing impaired performance in the presence of others (Quarter and Marcus, 1971; Newman et al., 1978; Wolf et al., 2015), a pattern that have been long demonstrated in adults, and which is known as social inhibition [Zajonc, 1965; Bond and Titus, 1983; see also Baron et al. (1978) and Sanders and Baron (1975) for negative effects of distraction on task performance]. Thus, presence of others seems to boost learning for children, which corroborates the “video deficit” effect: Direct interaction (presence of an observer) seems to generate social cues driving infant attention and motivating learning (Kuhl et al., 2003), resulting in better performance in direct interaction compared to video condition. On the contrary, direct interaction tends to hinder an adult’s performance, at least in difficult tasks. Following this line, a recent experiment revealed that students participating in a history lesson (provided by eyewitnesses of an historic period) and later tested on their insight in epistemological principles of history performed better when the lesson was provided in video than in live. The authors interpreted this “live deficit” effect as the result of a difficulty in building up the distance with the eyewitness needed for a critical approach to their accounts (Bertram et al., 2017). To summarize, adult performance in complex tasks is hindered in the presence of others. This social inhibition effect has been observed in several complex tasks such as complex reasoning, decision making, and theory of mind tasks for instance (see Zajonc, 1965; Bond and Titus, 1983). Here, we explored whether L2 vocabulary learning also suffers social inhibition with direct interaction.

### Present Study

To sum up, vocabulary learning has been deeply explored previously, but the social interaction aspect has been extensively explored only in toddlers. In the present study, we explored whether direct social interaction is a key factor in efficient vocabulary learning in adults. The question at hand is of major interest since more than 50% of the global population acquires a foreign language during adulthood. With the advent of technologies that we are facing, more and more teaching and learning strategies through media (without direct interaction) are proposed (Biocca et al., 2003). It is important to explore whether such learning tools are as efficient as traditional teaching with live (direct) interaction.

**FIGURE 1:**
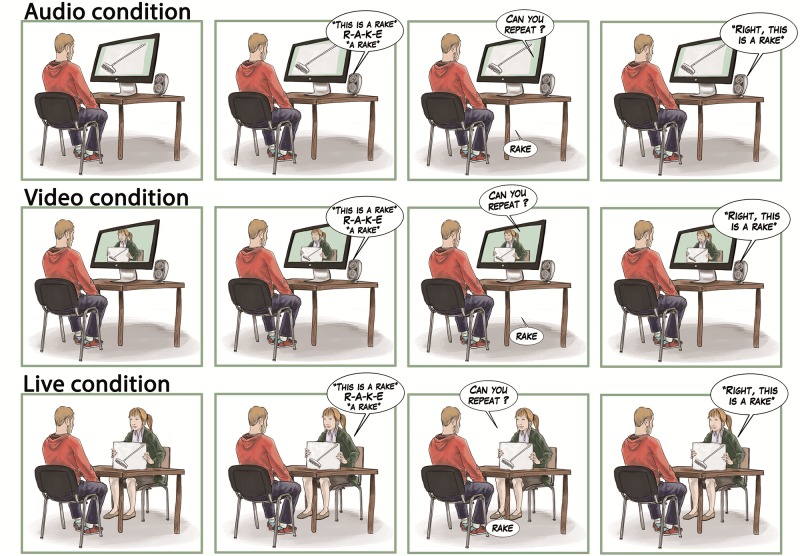
Description of the experimental procedure in each of the three learning conditions (NoFace, Video, and Live).

In a first experiment, we designed an L2 vocabulary learning experiment in which Spanish–English late bilinguals were exposed to three learning conditions (Figure 1). In the *Video condition*, participants were alone in the testing room. They were watching on a computer screen a teacher showing them objects while uttering and spelling the English name of each object. In the *Live condition*, participants were directly facing the teacher (physically present in the room) showing them the objects while uttering and spelling their names. Thus, in comparison to the *Video condition*, teacher’s presence in the room was added in the *Live condition*, in order to explore the influence of direct interaction on learning. In order to evaluate the direction of the social interaction effect (being detrimental or facilitatory), we added a baseline condition with no display or presence of the teacher (*NoFace condition*): Participants were alone in the testing room and were presented with objects displayed one-by-one on a computer screen, accompanied by the voice (but not the face) of the teacher uttering and spelling their names. Thus, there was no interaction in the *NoFace condition*, indirect interaction in the *Video condition*, and direct interaction in the *Live condition*. We compared accuracy in vocabulary learning in those three conditions.

In a second experiment, we replicated the design with Spanish–English late bilinguals exposed to the same three learning conditions. The first main goal was to replicate Experiment 1 and the second one was to generalize the results to L1 vocabulary learning. Consequently, participants had to learn new labels associated to new objects (instead of real objects as in Experiment 1). For direct comparison of the language effect, participants were tested both in L1 and L2 learning.

## Experiment 1

### Introduction

In Experiment 1, Spanish–English late bilinguals were asked to learn new L2 vocabulary in three different learning conditions: the *NoFace condition* (baseline), the *Video condition*, and the *Live condition*.

First, by comparing the *NoFace* and *Video* conditions, we expected performance to be higher in the latter because of the presence of the face with direct gaze. Encountering a face with a direct gaze orients attention to facial information (for a review, see Senju and Johnson, 2009) and facilitates information encoding (Fry and Smith, 1975; Sherwood, 1988; Strick et al., 2008). Interestingly for our purpose, Fullwood and Doherty-Sneddon (2006) tested the importance of direct gaze through video-mediated communication. They showed that more semantic information was recalled when provided by a speaker with directed compared to undirected gaze, the speaker being presented through television. This study suggests that the relevance of face display and direct gaze in adults during learning is high, even when learning is video-mediated. Thus, the presence of the face with a direct gaze in the *Video* condition might improve adults’ behavior on associated objects (here, L2 label learning), compared to the absence of a face in the baseline *NoFace* condition.

Regarding the influence of direct interaction (i.e., when comparing the *Video* and *Live* conditions), we considered two alternative hypotheses: While there are obvious benefits to learning via video (convenience, cost, and the ability to repeat lessons), previous research suggests that students find it more difficult to pay attention to lecture material when communication takes place over video (Candarli and Yuksel, 2012). Based on this previous observation, we might expect a “video deficit” effect in L2 adult learners (i.e., better learning in *Live* compared to *Video* condition), such as in children. On the contrary, the pattern observed in adults might be reversed (i.e., worse performance in *Live* compared to *Video* condition) because of social inhibition (impaired performance in the presence of others; Zajonc, 1965; Bond and Titus, 1983; see also Bertram et al., 2017).

### Materials and Methods

#### Participants

In Experiment 1, we tested 36 Spanish natives living in Spain, who were all University students at testing time and were late learners of English (see Table 1 for participant characteristics). They were asked to self-rate their language proficiency on a 10-point scale (“1” = low level of proficiency – “10” = native-like level). A self-assessed index (averaged for speech comprehension, speech production, reading, and writing) was measured in L1 (Spanish) and in L2 (English). Participants also performed a picture naming task to objectively assess their vocabulary knowledge. They were presented with 65 pictures that they had to name first in L1 and then in L2, and objective proficiency scores were measured in each language. All participants gave written informed consent and were paid to take part in the study that was approved by the BCBL Ethics Committee. Participant number was defined based on proper condition counterbalancing. Two participants were removed from analyses since they did not perform the learning task appropriately [no word properly learned across the entire experiment (three conditions)].

**TABLE 1:** Participant characteristics in Experiments 1 and 2.

	**Experiment 1**	**Experiment 2**
Sample	36	36
Age	24.0 (3.4)	22.1 (2.6)
	[19–29]	[19–30]
Eng-AoA	7.0 (2.1)	7.3 (3.3)
	[5–15]	[3–20]
Subj Eng-Prof	4.8 (1.9)	5.3 (1.4)
Obj Eng-Prof	29.2 (8.7)	32.6 (7.6)
Subj Sp-Prof	9.5 (1.0)	9.5 (0.8)
Obj Sp-Prof	64.6 (0.5)	64.5 (0.7)

*Sample, mean age (Age), age of acquisition of English (Eng-AoA), subjective and objective English proficiency (Subj and Obj Eng-Prof), subjective and objective Spanish proficiency (Subj and Obj Sp-Prof). Values reported are averages, standard deviations are in brackets, and ranges within square brackets.*

#### Material

Stimuli consisted of 60 English words that were low frequent (to assure that participants with medium level of English would most likely not know them), one or two syllables long, and highly concrete (see Appendix B for examples of stimuli). Spanish–English cognates, homophones, false friends, and polysemous words were excluded. Those English words were known by a maximum of 20% of the participants involved in a pre-test (10 Spanish–English bilinguals having a similar language history than the participants recruited afterward for the experiment, and who were asked the English translation for each Spanish word equivalent). Sixty black-and-white drawings or pictures referring to the words were selected [from the Snodgrass database (Snodgrass and Vanderwart, 1980) when available and from the web]. A second pre-test (10 Spanish natives asked to name each picture in Spanish) guaranteed high picture name agreement (98.2 ± 0.05%).

The 60 English words were divided into 5 lists of 12. The lists were matched for English frequency, number of letters, number of phonemes, number of syllables, imageability, familiarity, and concreteness, and for the equivalent variables in Spanish [all *p*s > 0.09; “MRC Psycholinguistic Database” used for English words (Coltheart, 1981) and “EsPal Database” for Spanish translations (Duchon et al., 2013)]. For each subject, three of the lists were presented (in each of the three conditions) in a randomized order. The 60 pictures were printed on thick A4-sized cards, used by the teacher for object presentation during the *Video* and *Live* conditions, and were included in a slide show displayed, together with the teacher’s voice, during the *NoFace* condition.

#### Procedure

Each participant was presented with three learning blocks, corresponding to three learning conditions (*NoFace*, *Video*, and *Live* conditions) with the same English native teacher (Figure 1). The teacher was a 25-year-old woman speaking with a neutral voice and with constant eye-contact. In the *Video* and *Live* conditions, the teacher was acting similarly, for each new word to learn: The teacher first showed a printed picture of the object (e.g., drawing of a rake). The teacher then said “This is a rake” and spelt the word before repeating “A rake.” The teacher then asked the participant to repeat the word aloud. This label repetition was included in the procedure to elicit active participation of the learner, which has been shown to boost learning (Troseth et al., 2006; Roseberry et al., 2014). Finally, the teacher said “Right, this is a rake” before putting down the image and moving on the next word (next image), until the end of the list. Physical aspect, neutral prosody, and constant eye-contact were similar in both conditions to make sure that the only difference between the two conditions was in the presence (or not) of the teacher in the room. In the *NoFace* condition, the teacher’s speech presented to the participant was equivalent to the other conditions, and the participant also had to repeat each word aloud. Thus, the *NoFace* condition was strictly similar to the *Video* condition regarding speech uttered. The only difference between those two conditions was in the visual display accompanying the audio: The teacher presenting the objects one by one was displayed in the *Video* condition while a simple slide show was presented together with the teacher’s voice in the *NoFace* condition. In the *NoFace* and *Video* conditions, the teacher left the room for the duration of the video. Word lists and conditions were counterbalanced across participants.

At the very beginning, participants were welcomed by the teacher (experimenter) speaking to them in Spanish. The teacher explained the task, after what she did not interact more with the participants (except in spelling the words in the *Live* condition and in asking participants to name the objects in the recall tests). Participants were instructed that they would have to learn 12 new English words, in three different teaching conditions, and that they would have to perform a recall test at the end of each condition. During each recall test (at the end of each teaching condition), the teacher (present in the room) presented the participants with each of the 12 pictures again (in the same order than during learning) and asked them to name each object in English. Participants’ responses were recorded and the teacher measured the number of words properly learned. Words were considered learned without considering proper native-like pronunciation. The number of words properly learned in each condition was submitted to a repeated-measures analysis of variance (ANOVA) with one 3-level factor (conditions *NoFace*, *Video*, and *Live*). The analysis by participant (F1 analysis hereafter) was an ANCOVA, several learners’ characteristics being entered as covariates: gender, age, age of acquisition of English, subjective and objective English proficiency, and subjective and objective Spanish proficiency. The analysis by item (F2 analysis hereafter) was an ANOVA.

At the end of the experiment (after the three counterbalanced blocks), the participants were asked if any of the words they had learnt were familiar to them before the experiment (i.e., they did not have to learn them because they already knew the English label beforehand). This happened for six participants: Four participants already knew one of the words and two participants already knew two of the words beforehand. Those eight items were removed from analyses (one from the *NoFace* condition, four from the *Video* condition, and three from the *Live* condition). Note that the results were identical when analyzing uncorrected learning scores.

### Results and Discussion

The ANOVA revealed a significant effect of condition [F1: *F*(2,25) = 5.47, *p* = 0.011, η^2^ = 0.30; F2: *F*(2,58) = 7.6, *p* = 0.001, η^2^ = 0.21]. Paired comparisons corrected for multiple comparisons (Bonferroni correction) revealed that significantly more words were learned in the *Video* condition than in the two other conditions (F1: Video–NoFace, *p* = 0.035; Video–Live, *p* = 0.010; F2: Video–NoFace, *p* = 0.02; Video–Live, *p* = 0.001). Learning did not significantly differ in the *NoFace* and *Live* conditions (*p* = 0.99 in F1 and F2 analyses; see Table 2 first row).

**TABLE 2:** Averaged percentage of labels learned (standard deviations into bracket) in each of the three learning conditions (NoFace, Video, and Live).

	**NoFace**	**Video**	**Live**
English – Experiment 1	23.5 (16.6)	32.8 (21.7)^*^	21.1 (15.9)
English – Experiment 2	22.6 (18.0)	30.9 (15.8)^*^	16.8 (10.7)
Spanish – Experiment 2	25.6 (20.3)	36.8 (20.1)^*^	19.1 (15.2)

*English word learning in Experiment 1 (first row), English label learning in Experiment 2 (second row), Spanish label learning in Experiment 2 (third row). Asterisks indicate the condition significantly differing from the other two, in each experiment.*

Further analyses revealed that participants’ gender did not significantly affect their behavior [main effect of gender: *F*(1,32) = 2.24, *p* = 0.14, η^2^ = 0.07; condition × gender interaction: *F*(1,32) = 1.14, *p* = 0.29, η^2^ = 0.03]. Moreover, none of the available participants’ characteristics significantly correlated with performance (see Appendix A, Table A1).

The first important result is that participants learned significantly more words in the *Video* than in the *NoFace* condition. This result shows that, as expected, the visual presentation of the teacher with direct gaze improves learning scores, compared to the presentation of only the voice of the teacher. This result supports the claim that a face display with direct gaze plays a fundamental role in social communication in adults (Fry and Smith, 1975; Sherwood, 1988; Strick et al., 2008; Senju and Johnson, 2009) and extends it to social situations such as vocabulary learning. This result also supports previous studies suggesting a high relevance of directed gaze during video-mediated learning in adults (Fullwood and Doherty-Sneddon, 2006). Note that this facilitation in learning in *Video* versus *NoFace* condition is even more striking when considering the whole learning environment: In the *Video* condition, pictures of objects were smaller on the screen, and there were several visual distractors (whole upper-part teacher’s body and background) attracting participants’ attention away from the pictures. Previous studies revealed that when participants see the face of the teacher (compared to the face hidden) they pay less attention to the task area (Kizilcec et al., 2014; Van Wermeskerken and Van Gog, 2017). Here, in a similar fashion, we can assume that less attention was paid to the pictures presented when the face of the teacher was visible (*Video* condition). Note that this lower level of attention is only speculative since no eye-movement recording was performed to monitor participant’s attention. Despite this lower level of attention to the task, participants performed better in the *Video* relative to *NoFace* condition. Interestingly, this is in line with previous research showing that seeing the face of the instructor during learning from video affects participant’s attention without modulating learning performance (Van Wermeskerken and Van Gog, 2017; Van Wermeskerken et al., 2017). In line with these studies, performance in the *Video* condition was not hindered by the teacher’s face display. Learning scores were even significantly higher, which reveals a significantly positive effect of teacher’s display with direct gaze during learning sessions. Since no gaze guidance was provided in the *Video* condition (the teacher was continuously looking at the camera during video recording), we can assume that better performance in *Video* relative to *NoFace* condition was triggered by attention and motivation (Kizilcec et al., 2014).

The second important result is that participants did not suffer from a “video deficit” effect during vocabulary learning. On the contrary, they actually learned fewer words in the *Live* than in the *Video* condition, suggesting that they suffered from social inhibition when the teacher was present in the room with them (Zajonc, 1965; Sanders and Baron, 1975; Baron et al., 1978; Bond and Titus, 1983). Note that we interpret the result pattern according to the social inhibition account, despite the fact that the teacher was not simply an observer, since he was actively involved in the task process. The wide literature on social inhibition tends to suggest that the presence of a passive observer or the presence of a judge (uttering positive or negative comments) both lead to a decrease in performance. In the present study, we show that the presence of a teacher (active observer but not judge) also negatively affects adult’s performance in the learning task. The presence of the teacher did not motivate learning (as it is the case for children; Kuhl et al., 2003), but hindered adults’ performance. This result is in line with a recent study showing that, despite self-evaluated lower interest and success, students perform better in some aspect of factual knowledge retention when taught through video than in live (Bertram et al., 2017).

The surprising opposite effect observed in adults compared to the “video deficit” effect repeatedly observed in children opens a new question on why adults suffer social inhibition with direct interaction, when children do not. The most evident reason is that adults in the present experiment were learning new vocabulary in their second language, while previous studies in children explored vocabulary learning in L1^1^. This major difference might explain the opposite pattern of results, because cognitive processes involved in L1 and L2 task performance vary drastically (see for instance Green, 1998; Clahsen and Felser, 2006). Secondly, adults in Experiment 1 were learning new words associated to objects that were already known and already had a name in L1. Children in previous experiments were learning new labels associated to new objects. Thirdly, the teacher in the present study was an English native speaker, meaning that she was an out-group member for the participants: She was speaking English with a native accent and Spanish with a foreign accent, while the opposite was true for the participants. It is known that task performance is hindered when facing an out-group compared to an in-group member (Tajfel et al., 1971; Brewer, 1999). Adults, but also infants and children, demonstrate social preference for members of their own native language group compared to members of another language group (Kinzler et al., 2007). This social disfavor for out-group members might be the cause of social inhibition in the present experiment.

Finally, note that none of the participants’ characteristics available (age, gender, age of acquisition, and proficiency) played a significant role in learning. Previous studies on vocabulary acquisition revealed significant effects of age and proficiency [Kojic-Sabo and Lightbown, 1999; Llanes and Muñoz, 2012; see Singleton and Ryan (2004) for a review]. In the present study, those variables did not play a significant role probably because of the low variability across participants (all participants were young adults, late, and low-proficient bilinguals in English). Interestingly, the lack of gender effect in learning is in line with previous studies revealing that neither the gender of the “model” nor the gender of the participant affected learning performance (Hoogerheide et al., 2016, 2017).

## Experiment 2

### Introduction

In order to better understand social inhibition in L2 vocabulary learning in adults, we designed a second experiment, in which participants had to learn new labels associated to new objects, facing an in-group member teacher (to mimic children’s learning conditions). For direct comparison of the language effect, participants were tested both in L1 and L2 learning.

Participants were presented with two consecutive sessions of three learning blocks each. They had to learn new labels in *NoFace*, *Video*, and *Live* conditions. They were taught English labels in one session and Spanish labels in the other session. English and Spanish labels were English-like and Spanish-like pseudowords associated with pictures of novel objects. Pseudowords and novel objects were used in this experiment (and not real words and/or real objects) to make sure participants were learning the association between new objects and their label in L1, as it is the case for children learning new vocabulary. Label learning in English session was similar to Experiment 1, with one main difference: The teacher in Experiment 2 was Spanish native, thus being an in-group member for the participants (i.e., speaking Spanish with a native accent and English with a Spanish accent, such as the participants). We expected to replicate the results’ pattern of Experiment 1 if social inhibition was due to the presence of the teacher in the room. On the contrary, if social inhibition in Experiment 1 was mainly due to the out-group member status of the teacher (her being English native and not Spanish native), we should observe better performance in *Live* than *Video* condition in Experiment 2, revealing the advantage of direct interaction with an in-group member teacher (as previously shown in children; Krcmar et al., 2007; DeLoache et al., 2010; O’Doherty et al., 2011; Roseberry et al., 2014). Label learning in Spanish session mimicked learning situations previously explored in children. If the pattern observed in Experiment 1 was caused by task performance in L2, we should observe here better performances in *Live* than *Video* condition, as previously observed in children performing learning tasks in L1. On the contrary, we hypothesized that the “social inhibition pattern” might be replicated in Spanish (L1) if adults suffer from social inhibition in vocabulary learning situations, whatever the language at hand.

### Materials and Methods

#### Participants

Thirty-six Spanish–English late bilinguals took part in Experiment 2. They were all Spanish natives living in Spain, University students at testing time, and later learners of English (see Table 1 for participant characteristics). All participants gave written informed consent and were paid to take part in the study that was approved by the BCBL Ethics Committee. Two participants were removed from analyses since they did not perform the learning task appropriately [no word properly learned across the entire experiment (three conditions)].

#### Material

Stimuli consisted of 30 English-like pseudowords and 30 Spanish-like pseudowords (see Appendix B for examples of stimuli). All pseudowords were four-to-six letter long [5.3(0.7) on average for English-like pseudowords and 5.2(0.6) for Spanish-like pseudowords; *t*-test: *p* = 0.69]. In order to mimic vocabulary learning in children (who have small vocabulary, i.e., novel words have very few neighbors), pseudowords had very few neighbors [0–3; 1.4(1.2) on average for English-like pseudowords and 1.4(1.0) for Spanish-like pseudowords; *t*-test: *p* = 0.99]. There was no pseudoword with any high frequent neighbor [neighbor frequency <60 per million; “Speech & Hearing Lab Neighborhood Database” used for English-like pseudowords (based on Hoosier Mental Lexicon Database; Nusbaum et al., 1984) and “BuscaPalabras” used for Spanish-like pseudowords (Davis and Perea, 2005)]. All pseudowords were checked by natives (three in each language) to ensure that they were easily pronounceable, could perfectly be real English/Spanish words, and were not very similar to real words. All Spanish-like pseudowords were treated as masculine in order to avoid extra gender learning in Spanish, which does not apply to English. The 30 English-like pseudowords were divided into 3 lists of 10, as well as the 30 Spanish-like pseudowords resulting in 6 lists of 10 pseudowords. English-like and Spanish-like lists were matched for pseudoword length and number of neighbors (all *p*s > 0.70). Sixty novel objects were selected from the NOUN Database (Horst and Hout, 2016) and were divided into 6 lists of 10 (see Appendix B for examples of stimuli). Visually similar objects were split in different lists. The six lists were matched for object familiarity, name ability, and for color and texture saliency [see ratings in Horst and Hout (2016); all *p*s > 0.39]. Each pseudoword list was assigned an object list and each pseudoword was paired with a novel object inside each list. The 60 novel object pictures were printed on thick A4-sized cards, used by the teacher for object presentation during the *Video* and *Live* conditions, and were included in a slide show displayed, together with the teacher’s voice, during the *NoFace* condition.

#### Procedure

Each participant was presented with two sessions of three learning conditions (Spanish and English session, order counterbalanced across participants). The teacher was a 20-year-old woman, Spanish–English late bilingual (as participants were). Teacher’s behavior in the three conditions was identical to Experiment 1, with the exception that she was addressing the participants in Spanish or in English depending on the session. Participants had to learn 10 new labels per condition, and word lists and conditions were counterbalanced across participants.

Participants were instructed that they would have to learn new words, in three different teaching conditions, first in English and then in Spanish (or vice versa). Apart from this modification, instructions and recall test were strictly identical to Experiment 1.

Participants were tested for proper learning at the end of each block (recall test), and the number of labels properly learned in each condition were submitted to a 2 × 2 × 3 repeated-measures ANOVA (two session orders – starting with Spanish or English session; two languages of learning – Spanish or English; three learning conditions – *NoFace*, *Video*, and *Live*). The analysis by participant (F1 analysis hereafter) was an ANCOVA, learners’ characteristics being entered as covariates: gender, age, age of acquisition of English, subjective and objective English proficiency, and subjective and objective Spanish proficiency. The analysis by item (F2 analysis hereafter) was an ANOVA.

### Results and Discussion

The ANOVA revealed a significant effect of condition [F1: *F*(2,23) = 25.95, *p* < 0.001, η^2^ = 0.69; F2: *F*(2,57) = 18.49, *p* < 0.001, η^2^ = 0.39], and no other main effects or interactions reached significance [language effect: *p* = 0.093, η^2^ = 0.09 and *p* = 0.26, η^2^ = 0.02 for F1 and F2 analyses, respectively; language × condition interaction: *p* = 0.72, η^2^ = 0.02 and *p* = 0.77, η^2^ = 0.009, respectively; session effect: *p* = 0.99, η^2^ = 0.00; language × session interaction: *p* = 0.42, η^2^ = 0.02; condition × session interaction: *p* = 0.53, η^2^ = 0.04; condition × language interaction: *p* = 72, η^2^ = 0.02; triple interaction: *p* = 0.46, η^2^ = 0.05]. Paired comparisons corrected for multiple comparisons (Bonferroni correction) revealed that significantly more labels were learned in the *Video* condition than in the two other conditions (F1: Video–NoFace, *p* = .012; Video–Live, *p* < 0.001; F2: Video–NoFace, *p* = 0.001; Video–Live, *p* < 0.001). Learning did not significantly differ in the *NoFace* and *Live* conditions (*p* = 0.072 in F1 and *p* = 0.06 in F2 analyses; see Table 2, second and third rows)^2^.

Further analyses revealed that participants’ gender did not significantly affect their behavior [English label learning: main effect of gender: *F*(1,32) = 1.70, *p* = 0.20, η^2^ = 0.05; condition × gender interaction: *F*(1,32) = 0.41, *p* = 0.53, η^2^ = 0.01; Spanish label learning: *F*(1,32) = 0.47, *p* = 0.50, η^2^ = 0.01; condition × gender interaction: *F*(1,32) = 0.96, *p* = 0.33, η^2^ = 0.03]. Moreover, none of the available participants’ characteristics significantly correlated with performance (see Appendix A, Table A2 for English and Table A3 for Spanish label learning).

We replicated the beneficial effect of the face display with direct gaze in learning, by showing that both in English and Spanish, participants learned more labels in *Video* compared to *NoFace* condition. More importantly, we also replicated the detrimental effect of the presence of the teacher (direct interaction), by showing that participants learned fewer labels in the *Live* compared to the *Video* condition, both in English and Spanish. Thus, it seems that social inhibition hindering vocabulary learning in the *Live* condition is not due to the fact that the task is performed in L2, neither to the fact that the teacher is a “foreign language group” member.

As in Experiment 1, none of the participants’ characteristics available (age, gender, age of acquisition, and proficiency) played a significant role in learning, probably because the range in variability in those variables was very low, since it was not the main focus of the study. Thus, no novel important observation can be added to research on how individual characteristics interplay with vocabulary acquisition.

## General Discussion

Results from Experiments 1 and 2 suggest that the presence of the teacher in the room during a vocabulary learning session (i.e., direct interaction) provokes social inhibition and consequently a decrease in adults’ performance (compared to a similar teaching procedure video-mediated). Such social inhibition hinders performance when learning L1 and L2 new vocabulary, and when the teacher is an in-group or an out-group member, suggesting a domain-general inhibiting effect independent from some important linguistic and social factors.

This intriguing result reveals that new vocabulary learning differs in adults and children, not only because of fundamental differences in language knowledge such as vocabulary size and processing automaticity (see for instance Hahn and Gershkoff-Stowe, 2010; Snedeker et al., 2012; Gershkoff-Stowe and Hahn, 2013), but also because of social factors such as direct interaction. Interestingly, a recent experiment showed that children and adults do not similarly benefit from studying abroad (i.e., learning context; Llanes and Muñoz, 2012). This study on learning context and our present set of experiments together tend to suggest that social factors (such as learning context, direct interaction) do not necessarily affect children and adults in the same way.

This is the first study, to our knowledge, showing that adults suffer social inhibition during learning of vocabulary lists with direct interaction. This has potential important consequences for learning procedures. In fact, video-mediated teaching might not be detrimental for adults learning lists of vocabulary in a second language. This is not to say that video-mediated teaching should replace classroom environment (which provides obvious advantages) but that benefits to learning via video (price, repetition, etc.) could be used, for instance, for vocabulary list acquisition. Importantly, those video-mediated programs should take into account the positive effect of directed gaze during learning session, meaning that the most efficient vocabulary learning should be obtained via video-mediated tools including a teacher’s “display” and directed gaze (Fullwood and Doherty-Sneddon, 2006).

Since we report here the first study comparing *Live* and *Video* conditions in vocabulary learning in adults, this study has to be further replicated and extended for generalization. Moreover, several important variables should be manipulated in further studies, since we tested here a very limited teaching situation: Learners are usually exposed to new words more than once, and not only in isolation but also in sentence context. Learners also usually know their teachers (who do not behave neutrally) and extensively interact with them [see Schmitt (2008); Pinar (2016), and Rasouli and Jafari (2016) for reviews]. Further studies should also explore similar teaching situations in another adult population (to generalize to other bilinguals than Spanish–English late bilinguals tested in the present study), as well as long-term effects of learning (and not only immediate recall as it is the case here). Our study opens several other interesting questions such as whether social inhibition induced by direct interaction can be canceled when teaching environment and teacher are familiar, and whether the effects would be similar with different types of learning and recall. For instance, the type of recall test employed in this study was highly challenging because of its difficulty (free naming) and stressing environment (presence of the teacher). It might be the case that social inhibition is lower (or even reversed) when the recall task is easier (i.e., recognition task) and/or the teacher is absent during the recall session. Finally, other teaching situations such as without active participation during learning (having to repeat the word out loud), or without having to articulate the name of the picture during recall, should be tested in order to get a clear picture of what is the optimal teaching situation for adult late learners of a vocabulary list in a second language. Strong conclusions cannot be drawn at this stage, but the present study reports a first set of interesting results showing that video-mediated programs might be beneficial for efficient learning of vocabulary lists in adults.

Regarding the research field on vocabulary acquisition, the present study provides novel important information on learning environments: In line with previous studies exploring learning through video (Fullwood and Doherty-Sneddon, 2006; Bertram et al., 2017), we showed that a simple task like new label learning can be achieved efficiently through video with teacher’s display with direct gaze. Regarding other factors known to affect vocabulary learning (e.g., age and proficiency; Singleton and Ryan, 2004), the present study does not provide new information given that none of the available participants’ characteristics played a significant role in learning, and given that important variables such as motivation, strategy used in learning, mental effort, and perceived competence (Schmitt, 2008; Hoogerheide et al., 2016; Pinar, 2016; Rasouli and Jafari, 2016) were not assessed in the present study. Even if based on a null result, the present study provides further evidence for a lack of effect of model and participant gender in learning performance (Hoogerheide et al., 2016, 2017).

## Data Availability

The datasets generated for this study are available on request to the corresponding author.

## Ethics Statement

This study was carried out in accordance with the recommendations of the BCBL Ethics Committee with written informed consent from all subjects. All subjects gave written informed consent in accordance with the Declaration of Helsinki. The protocol was approved by the BCBL Ethics Committee.

## Author Contributions

CM and NM developed the study concept and designed the study. AU performed the testing and data collection. AU and CM performed the data analysis. All authors took part in data interpretation and approved the final version of the manuscript for submission. CM drafted the manuscript, and NM and AU provided critical revisions.

## Conflict of Interest Statement

The authors declare that the research was conducted in the absence of any commercial or financial relationships that could be construed as a potential conflict of interest.

## Footnotes

1 Note that adults and children highly differ also on vocabulary size and word retrieval automaticity, which makes learning sensitive to general development (see Hahn and Gershkoff-Stowe, 2010). Nevertheless, there is no clear reason why those factors might affect NoFace-, video-, or live-mediated learning in a different manner.

2 An extra analysis comparing directly English word learning in Experiments 1 and 2 confirmed that the pattern of result was strictly identical in the two experiments since there was no experiment × condition interaction [*F*(2,65) = 0.20, *p* = 0.82].

## Appendix A

**TABLE A1:** Correlation analyses between participants’ characteristics and performance in learning task in Experiment 1.

	**NoFace**	**Video**	**Live**
Age	−0.036 *0.84*	0.045 *0.80*	0.108 *0.54*
Eng-AoA	0.169 *0.34*	0.195 *0.27*	0.130 *0.46*
Subj Sp-Prof	0.137 *0.44*	0.194 *0.27*	−0.132 *0.46*
Subj Eng-Prof	−0.113 *0.53*	−0.181 *0.31*	−0.210 *0.23*
Obj Sp-Prof	−0.036 *0.84*	0.005 *0.98*	−0.003 *0.99*
Obj Eng-Prof	−0.202 *0.25*	−0.005 *0.98*	0.092 *0.60*

*Correlation scores (Pearson test) and significance *p*-values (in italic) are reported for each learning condition (NoFace, Video, and Live) and each participants’ characteristic [Age, age of acquisition of English (Eng-AoA), subjective and objective Spanish proficiency (Subj and Obj Sp-Prof), subjective and objective English proficiency (Subj and Obj Eng-Prof)].*

**TABLE A2:** Correlation analyses between participants’ characteristics and performance in learning task in Experiment 2 (English labels).

	**NoFace**	**Video**	**Live**
Age	0.138 *0.44*	−0.257 *0.14*	0.315 *0.07*
Eng-AoA	0.091 *0.61*	−0.223 *0.21*	0.087 *0.63*
Subj Sp-Prof	−0.116 *0.52*	−0.077 *0.67*	0.283 *0.10*
Subj Eng-Prof	−0.161 *0.38*	0.010 *0.96*	0.157 *0.39*
Obj Sp-Prof	−0.141 *0.43*	−0.241 *0.17*	0.007 *0.97*
Obj Eng-Prof	0.094 *0.60*	0.106 *0.55*	0.010 *0.95*

*Correlation scores (Pearson test) and significance *p*-values (in italic) are reported for each learning condition (NoFace, Video, and Live) and each participants’ characteristic.*

**TABLE A3:** Correlation analyses between participants’ characteristics and performance in learning task in Experiment 2 (Spanish labels).

	**NoFace**	**Video**	**Live**
Age	0.016 *0.93*	−0.323 *0.07*	−0.075 *0.67*
Eng-AoA	−0.078 *0.66*	−0.269 *0.12*	−0.276 *0.11*
Subj Sp-Prof	−0.075 *0.68*	−0.044 *0.81*	−0.015 *0.94*
Subj Eng-Prof	−0.190 *0.30*	−0.209 *0.25*	0.124 *0.50*
Obj Sp-Prof	0.061 *0.73*	−0.097 *0.59*	0.091 *0.61*
Obj Eng-Prof	0.295 *0.09*	0.086 *0.63*	0.208 *0.24*

*Correlation scores (Pearson test) and significance *p*-values (in italic) are reported for each learning condition (NoFace, Video, and Live) and each participants’ characteristic.*

## Appendix B

**TABLE B1:** Examples of stimuli used in Experiment 1 (English words and pictures of associated objects) and in Experiment 2 (English and Spanish pseudowords and pictures of novel objects).

**Experiment 1**		**Experiment 2**		
**English words**	**Pictures**	**English pseudowords**	**Spanish pseudowords**	**Pictures**
Crank		Clisp	Astivo	
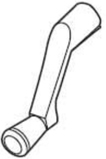
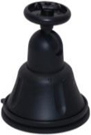
Clog		Cresk	Bereo	
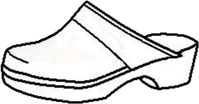
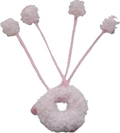
Rake		Cenon	Lisde	
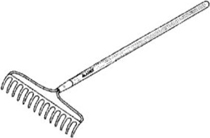
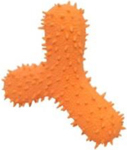
Sickle		Soople	Nilto	
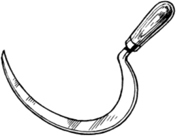
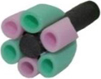
Whisk		Drall	Panio	

## References

[B1] Anderson D. R. Pempek T. A. (2005). Television and very young children. *Am. Behav. Sci.* 48 505–522. 10.1177/0002764204271506

[B2] Baron R. S. Moore D. Sanders G. S. (1978). Distraction as a source of drive in social facilitation research. *J. Pers. Soc. Psychol.* 36:816 10.1037//0022-3514.36.8.816

[B3] Barr R. Hayne H. (1999). Developmental changes in imitation from television in infancy. *Child Dev.* 70 1067–1081. 10.1111/1467-8624.00079 10546335

[B4] Bertram C. Wagner W. Trautwein U. (2017). Learning historical thinking with oral history interviews: a cluster randomized controlled intervention study of oral history interviews in history lessons. *Am. Educ. Res. J.* 54 444–484. 10.3102/0002831217694833

[B5] Biocca F. Harms C. Burgoon J. K. (2003). Toward a more robust theory and measure of social presence: review and suggested criteria. *Pres. Teleoperat. Virt. Environ.* 12 456–482.

[B6] Bond C. F. Titus L. J. (1983). Social facilitation: a meta-analysis of 241 studies. *Psychol. Bull.* 94 265–292. 10.1037/0033-2909.94.2.265 6356198

[B7] Brewer M. (1999). The psychology of prejudice: ingroup love or outgroup hate. *J. Soc. Issues* 55 429–444. 10.1111/0022-4537.00126

[B8] Candarli D. Yuksel H. G. (2012). Students’ perceptions of video-conferencing in the classrooms in higher education. *Proc. Soc. Behav. Sci.* 47 357–361. 10.1016/j.sbspro.2012.06.663

[B9] Chevallier C. Parish-Morris J. Tonge N. Le L. Miller J. Schultz R. T. (2014). susceptibility to the audience effect explains performance gap between children with and without autism in a theory of mind task. *J. Exp. Psychol. Gen.* 143 972–979. 10.1037/a0035483 24392710 PMC4038654

[B10] Clahsen H. Felser C. (2006). How native-like is non-native language processing? *Trends Cogn. Sci.* 10 564–570. 10.1016/j.tics.2006.10.002 17071131

[B11] Coltheart M. (1981). The MRC psycholinguistic database. *Q. J. Exp. Psychol.* 33A 497–505. 10.1080/14640748108400805

[B12] Cook V. (2008). *Second Language Learning and Language Teaching*, 4th Edn London: Hodder Education.

[B13] Davis C. J. Perea M. (2005). BuscaPalabras: a program for deriving orthographic and phonological neighborhood statistics and other psycholinguistic indices in Spanish. *Behav. Res. Methods* 37 665–671. 10.3758/bf03192738 16629300

[B14] DeLoache J. Chiong C. Sherman K. Islam N. Vanderborght M. Troseth G. L. (2010). Do babies learn from baby media? *Psychol. Sci.* 21 1570–1574. 10.1177/0956797610384145 20855901

[B15] Dewey D. (2008). Japanese vocabulary acquisition by learners in three contexts. *Front. Interdiscip. J. Study Abroad* 15 127–148.

[B16] Duchon A. Perea M. Sebastián-Gallés N. Martí A. Carreiras M. (2013). EsPal: one-stop shopping for spanish word properties. *Behav. Res. Methods* 45 1246–1258. 10.3758/s13428-013-0326-1 23468181

[B17] Ellis R. (1985). *Understanding Second Language Acquisition.* Oxford: Oxford University Press.

[B18] Fry R. Smith G. F. (1975). The effects of feedback and eye contact on performance of a digit-coding task. *J. Soc. Psychol.* 96 145–146. 10.1080/00224545.1975.9923275 16081035

[B19] Fullwood C. Doherty-Sneddon G. (2006). Effect of gazing at the camera during a video link on recall. *Appl. Ergon.* 37 167–175. 10.1016/j.apergo.2005.05.003 16081035

[B20] Gershkoff-Stowe L. Hahn E. R. (2013). Word comprehension and production asymmetries in children and adults. *J. Exp. Child Psychol.* 114 489–509. 10.1016/j.jecp.2012.11.005 23270795

[B21] Green D. W. (1998). Mental control of the bilingual lexico-semantic system. *Biling. Lang. Cogn.* 1 67–81. 10.1017/s1366728998000133

[B22] Gu Y. (2005). Gender, academic major, and vocabulary learning strategies of Chinese EFL learners. *RELC J.* 33 35–54. 10.1177/003368820203300102

[B23] Hahn E. R. Gershkoff-Stowe L. (2010). Children and adults learn actions for objects more readily than labels. *Lang. Learn. Dev.* 6 283–308. 10.1080/15475441003635315

[B24] Hoogerheide V. Loyens S. M. M. van Gog T. (2016). Learning from video modeling examples: does gender matter? *Instr. Sci.* 44 69–86. 10.1007/s11251-015-9360-y

[B25] Hoogerheide V. van Wermeskerken M. van Nassau H. van Gog T. (2017). Model-observer similarity and task-appropriateness in learning from video modeling examples - do model and student gender affect test performance, self-efficacy, and perceived competence? *Comput. Hum. Behav.* 89 457–464. 10.1016/j.chb.2017.11.012

[B26] Horst J. S. Hout M. C. (2016). The novel object and unusual name (NOUN) database: a collection of novel images for use in experimental research. *Behav. Res. Methods* 48 1393–1409. 10.3758/s13428-015-0647-3 26424438

[B27] Kinzler K. D. Dupoux E. Spelke E. S. (2007). The native language of social cognition. *Proc. Nat. Acad. Sci. U.S.A.* 104 12577–12580. 17640881 10.1073/pnas.0705345104 PMC1941511

[B28] Kizilcec R. F. Papadopoulos K. Sritanyaratana L. (2014). “Showing face in video instruction: effects on information retention, visual attention, and affect,” in *Proceedings of the Annual SIGCHI Conference on Human Factors in Computing Systems* (New York, NY: Association for Computing Machinery).

[B29] Kojic-Sabo I. Lightbown P. M. (1999). Students’approaches to vocabulary learning and their relationship to success. *Mod. Lang. J.* 83 176–192. 10.1111/0026-7902.00014

[B30] Krcmar M. Grela B. G. Lin Y.-J. (2007). Can toddlers learn vocabulary from television? An experimental approach. *Med. Psychol.* 10 41–63.

[B31] Kuhl P. K. Tsao F.-M. Liu H.-M. (2003). Foreign-language experience in infancy: effects of short-term exposure and social interaction on phonetic learning. *Proc. Nat. Acad. Sci. U.S.A.* 100 9096–9101. 10.1073/pnas.1532872100 12861072 PMC166444

[B32] Llanes À Muñoz C. (2012). Age effects in a study abroad context: children and adults studying abroad and at home. *Lang. Learn.* 63 63–90. 10.1111/j.1467-9922.2012.00731.x

[B33] Meddock T. Parsons J. Hill K. (1971). Effects of an adult’s presence and praise on young children’s performance. *J. Exp. Child Psychol.* 12 197–211. 10.1016/0022-0965(71)90004-x

[B34] Nation P. (2001). *Learning Vocabulary in Another Language.* Cambridge: Cambridge University Press.

[B35] Newman A. Dickstein R. Gargan M. (1978). Developmental effects in social facilitation and in being a model. *J. Psychol.* 99 143–150. 10.1080/00223980.1978.9921454

[B36] Nusbaum H. C. Pisoni D. B. Davis C. K. (1984). *Sizing up the Hoosier Mental Lexicon: Measuring the Familiarity of 20,000 Words* Research on Speech Perception Progress Rep. No. 10. Bloomington, IN: Indiana University.

[B37] O’Doherty K. Troseth G. L. Shimpi P. M. Goldenberg E. Akhtar N. Saylor M. M. (2011). Third-party social interaction and word learning from video. *Child Dev.* 82 902–915. 10.1111/j.1467-8624.2011.01579.x 21418054 PMC3089674

[B38] Pinar A. (2016). Second language acquisition in a study abroad context: findings and research directions. *Colomb. Appl. Linguist. J.* 18 83–94.

[B39] Quarter J. Marcus A. (1971). Drive level and the audience effect: a test of Zajonc’s theory. *J. Soc. Psychol.* 83 99–105. 10.1080/00224545.1971.9919977 5100008

[B40] Rasouli F. Jafari K. (2016). A deeper understanding of L2 vocabulary learning and teaching: a review study. *Int. J. Lang. Linguist.* 4 40–46.

[B41] Roseberry S. Hirsh-Pasek K. Golinkoff R. M. (2014). Skype me! socially contingent interactions help toddlers learn language. *Child Dev.* 85 956–970. 10.1111/cdev.12166 24112079 PMC3962808

[B42] Sanders G. S. Baron R. S. (1975). The motivating effects of distraction on task performance. *J. Personal. Soc. Psychol.* 32 956–963. 10.1037/0022-3514.32.6.956

[B43] Schmitt N. (2008). Review article: instructed second language vocabulary learning. *Lang. Teach. Res.* 12 329–363. 10.1177/1362168808089921

[B44] Senju A. Johnson M. H. (2009). The eye contact effect: mechanisms and development. *Trends Cogn. Sci.* 13 127–134. 10.1016/j.tics.2008.11.009 19217822

[B45] Sherwood J. V. (1988). Facilitative effects of gaze upon learning. *Percept. Mot. Ski.* 64 1275–1278. 10.2466/pms.1987.64.3c.1275 16081035

[B46] Singleton D. Ryan L. (2004). *Language Acquisition*, 2nd Edn Bristol: Multilingual Matters Ltd.

[B47] Snedeker J. Geren J. Shafto C. L. (2012). Disentangling age and linguistic experience: a longitudinal study of the acquisition of English in internationally-adopted children. *Cogn. Psychol.* 65 39–76. 10.1016/j.cogpsych.2012.01.004 22417632

[B48] Snodgrass J. G. Vanderwart M. (1980). A standardized set of 260 pictures: norms for name agreement, image agreement, familiarity, and visual complexity. *J. Exp. Psychol. Hum. Learn.* 6 174–215. 10.1037//0278-7393.6.2.174 7373248

[B49] Stirling J. (2003). Helping students to learn the vocabulary that we teach them. *Lang. Teach. J.* 49 133–143.

[B50] Strick M. Holland R. W. van Knippenberg A. (2008). Seductive eyes: attractiveness and direct gaze increase desire for associated objects. *Cognition* 106 1487–1496. 10.1016/j.cognition.2007.05.008 17601526

[B51] Tajfel H. Billig M. G. Bundy R. P. Flament C. (1971). Social categorization and intergroup behaviour. *Eur. J. Soc. Psychol.* 1 149–178. 10.1002/ejsp.2420010202

[B52] Tennie C. Frith U. Frith C. (2010). Reputation management in the age of the world-wide web. *Trends Cogn. Sci.* 14 482–488. 10.1016/j.tics.2010.07.003 20685154

[B53] Troseth G. L. Saylor M. M. Archer A. H. (2006). Young children’s use of video as a source of socially relevant information. *Child Dev.* 77 786–799. 10.1111/j.1467-8624.2006.00903.x 16686801

[B54] Van Wermeskerken M. Ravensbergen S. Van Gog T. (2017). Effects of instructor presence in video modeling examples on attention and learning. *Comput. Hum. Behav.* 89 430–438. 10.1016/j.chb.2017.11.038

[B55] Van Wermeskerken M. Van Gog T. (2017). Seeing the instructor’s face and gaze in demonstration video examples affects attention allocation but not learning. *Comput. Educ.* 113 98–107. 10.1016/j.compedu.2017.05.013

[B56] Webb S. (2007). The effects of repetition on vocabulary knowledge. *Appl. Linguist.* 28 46–65. 10.1093/applin/aml048

[B57] Wolf L. K. Bazargani N. Kilford E. J. Dumontheil I. Blakemore S.-J. (2015). The audience effect in adolescence depends on who’s looking over your shoulder. *J. Adolesc.* 43 5–14. 10.1016/j.adolescence.2015.05.003 26043167 PMC4533226

[B58] Zajonc R. (1965). Social facilitation. *Science* 149 269–274. 14300526 10.1126/science.149.3681.269

